# Mixing due to Solution Switch Limits the Performance of Electrosorption for Desalination

**DOI:** 10.1021/acs.est.4c02681

**Published:** 2024-07-18

**Authors:** Weifan Liu, Longqian Xu, Zezhou Yang, Xudong Zhang, Shihong Lin

**Affiliations:** † Department of Civil and Environmental Engineering, Vanderbilt University, Nashville, Tennessee 37235-1831, United States; ‡ Department of Chemical and Bimolecular Engineering, Vanderbilt University, Nashville, Tennessee 37235-1831, United States

**Keywords:** desalination, electrosorption, solution switch, mixing, energy consumption

## Abstract

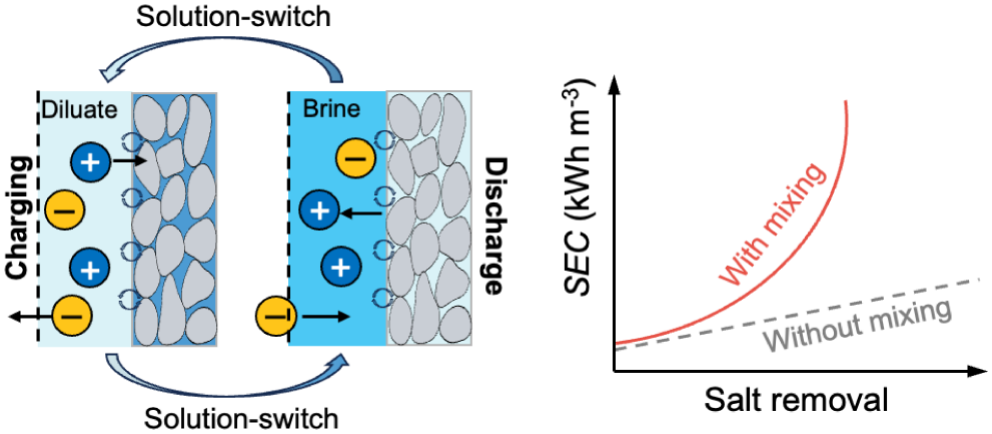

Electrosorption (ES) is a research frontier in electrochemical separation, with proven potential applications in desalination, wastewater treatment, and selective resource extraction. However, due to the limited adsorption capacity of film electrodes, ES requires short circuiting or circuit reversal, accompanied by a solution switch between the feed solution and receiving solution, to sustain desalination over many charge–discharge cycles. In previously reported studies, solution switches have been commonly ignored to simplify experimental procedures, and their impacts on separation performance are thus not well understood. This study aims to provide a quantitative analysis of the impacts of mixing due to a solution switch on the performance of ES-based desalination. A numerical model of ES has been employed to evaluate the adverse effects of the solution switch on the desalination performance in three commonly used operation modes. The analysis reveals that the impacts of mixing due to solution-switch are more severe with a larger concentration difference between the desalinated water and the brine and provides insights into the effectiveness of increasing electrode loading or specific capacity in mitigating the detrimental impacts of mixing. Even with state-of-the-art systems, producing freshwater from seawater or even brackish water with medium-to-high salinity is practically challenging due to the presence of solution switch.

## Abstract 2

Mixing due to solution switch compromises the performance of desalination using electrosorption.

## Introduction

Electrochemical separation stands as a burgeoning frontier in ion and molecular separation due to its absence of chemical use, operational flexibility, scalability, and wide-spectrum applicability.^1^ Electrosorption (ES) or capacitive deionization (CDI) is an important category of electrochemical separation inspired by the mechanism of battery and supercapacitor for energy storage.^1−3^ Extensive research has been performed to develop ES for desalination,^1,4,5^ water treatment,^6,7^ nutrient recovery,^8,9^ and resource extraction.^10,11^ ES, employing capacitive or Faradaic electrodes, adsorbs ions from aqueous solutions during charging and releases them upon voltage polarity reversal during discharging.^2,12^ Recent years have also seen a surge of interest in applications of ES beyond desalination,^13^ notably in direct lithium extraction (DLE) and nutrient recovery.^10,14,15^

The architectures of ES cells are predominantly based on film electrodes. These architectures include flow-by ES,^2,16^ flow-through ES,^16,17^ inverted ES,^18^ and those integrating ion exchange membranes (IEMs), such as membrane ES,^19,20^ rocking chair ES,^21^ and hybrid ES.^22^ Despite the difference in specific configurations, ES based on film electrodes shares a similar general working principle, i.e., ions are adsorbed from the feed solution to the electrodes in the charge half-cycle and stored in the electrode until saturation (for constant voltage operation) or the cell voltage becomes too high (for constant current operation), and then they are released to a receiving solution in the discharge half-cycle.^1,2,22^

**Figure 1:**
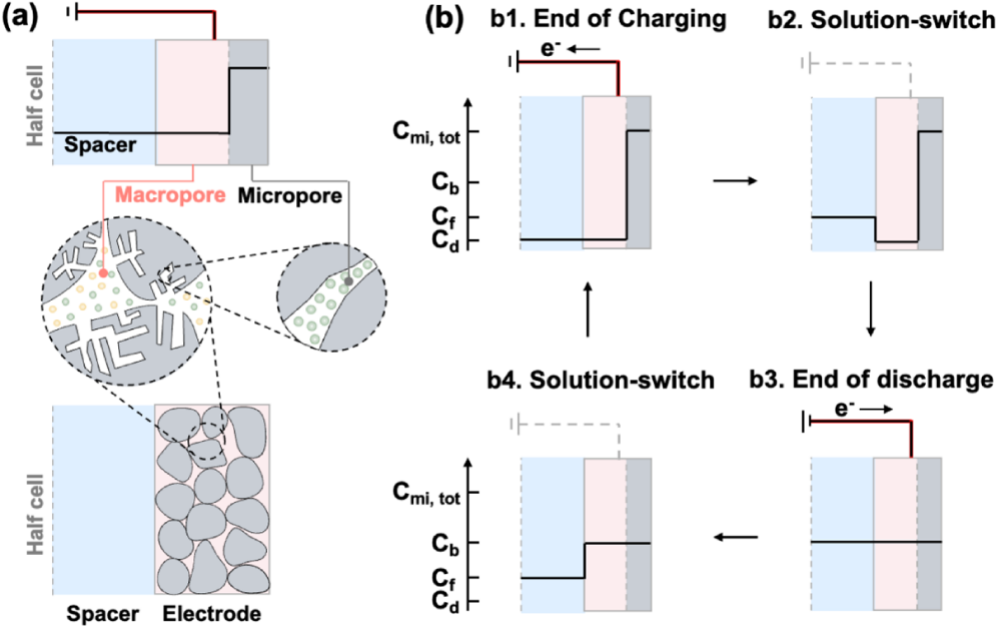
(a) Schematics of a typical ES half-cell comprising the spacer channel (i.e., flow channel) and the AC electrode with both macro- and micropores. The macropores and micropores occupy the same macroscopic electrode domain as in the bottom schematic. However, the top schematic intentionally splits the ES half-cell into three distinct domains to facilitate the semiquantitative representation of salt or ion concentrations in these three phases. Such a representation is used throughout Figure 1b. (b) Semiquantitative concentration profiles for different stages of a full charge–discharge cycle, including (b1) the end of charging, (b2) solution switch after charging and prior to discharging, (b3) the end of discharge, and (b4) solution switch after discharge and prior to charging. The black lines provide a semiquantitative representation of the concentration profiles with *c*_f_, *c*_b_, and *c*_d_ representing the salt concentrations of the feed, brine, and diluate streams, respectively. *c*_mi,tol_ is the total ion concentration in the micropores.

The ion storage sites are micropores in activated carbon (AC) electrodes^22^ and crystal lattice in intercalation electrodes.^12^ The space between particles of AC or intercalation materials is called macropores, which are typically saturated with the solution contacting the electrodes to promote fast ion transport (Figure 1a). All film-electrode-based ES configurations require physically switching the feed and the receiving solutions between the charge and discharge half-cycles, rendering the ES operation noncontinuous.^23,24^ A complete ES cycle includes the charge (adsorption) and discharge (desorption) half-cycles as well as two solution-switch steps between the two half-cycles (Figure 1b). These solution-switch steps impose critical limitations on the performance of ES.^25^ These limitations are likely to be recognized in the research community but have never been systematically investigated and quantified.

High-performance ES electrodes typically require excellent hydrophilicity and high porosity to facilitate fast ion transport between the solution and ion storage sites of the electrodes.^26,27^ Consequently, the solution switch in film electrode-based ES systems inevitably results in the mixing of residual solution within the macropores, with the new solution entering the flow channels (Figure 1b). Such mixing compromises the energy efficiency of ES and, in the case of selective ES, also undermines the separation selectivity.^25^ The phenomenon of solution mixing is also relevant to various electrochemical processes with a semibatch operation mode.^23,28^ In this work, we focus primarily on the adverse impacts of mixing on the performance of ES-based desalination using a typical cell architecture (i.e., we do not investigate its effect on ES-based selective separation).

The flow channels of the ES cell are alternately occupied by the diluate stream in the charge half-cycle and the brine stream in the discharge half-cycle. There are two instances of mixing due to solution switch in a complete ES cycle for desalination. At the end of the charge half-cycle, the salt concentration of solution in the electrode macropores equals that of the diluate (Figure 1, b1). Upon introduction of the receiving solution (at feed salinity), mixing occurs between the residual solution in the macropores with a low concentration and the receiving solution with a higher concentration (Figure 1, b2). At the end of the discharge half-cycle, the salt concentration in the macropores equals that of the brine (Figure 1, b3). The brine remaining in the electrode macropores mixes with the feed solution entering the flow channels, raising the concentration of the solution in the feed channel beyond the feed salinity at the start of the charge half-cycle (Figure 1, b4).

In this study, we perform numerical modeling to systematically evaluate the impacts of the solution switch on the performance of ES-based desalination. Specifically, we quantify the extent to which mixing due to solution switch compromises the ability of ES to produce freshwater under both single-pass and semibatch operation modes. We also investigate how the impacts of solution switch on salt removal and energy consumption depend on several key parameters, including feed salinity, water recovery, electrode macroporosity, loading, and capacity.

## Theory

### Modified Donnan Theory for the Salt Adsorption in Micropores

We apply the modified Donnan model (mD) to simulate salt adsorption and desorption in porous AC electrodes.^2,29^ The ions are mainly stored in the electrical double layers (EDLs) in the micropores of the porous AC electrode. The solution in the macropore has the same composition as the bulk solution, and charge neutrality is maintained for both macropores and the bulk solution. For 1:1 salt such as NaCl, the ion concentration in the micropores, *c*_mi,i_, relates to the salt concentration in the macropores, *c*_mA_, according to
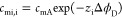
1where *z*_i_ is the ion valence and Δϕ_D_ is the Donnan potential between the micropores and macropores. The subscript “i” is ignored for *c*_mA_ for the symmetric salt due to charge neutrality in the macropores. For symmetric salt, the charge density, defined as the net charge in the micropores (σ_mi_ = ∑_i_*z*_i_*c*_mi,i_,*z*_i_ is the valence of species ″i″), can be expressed as
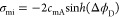
2

The total ion concentration in the micropores, *c*_mi,tot_ (*c*_mi,tot_ = ∑_i_*c*_mi,i_), can be obtained by combining eqs 1 and 2:
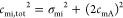
3

The micropore charge density, σ_mi_, can also relate to the Stern layer potential drop, Δϕ_s_, according to the following equation:
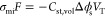
4where *F* is the Faraday constant, *C*_st,vol_ is the volumetric Stern layer capacitance (F L^–1^), and *V*_T_ is the thermal voltage (25.7 mV at 25 °C). *C*_st,vol_ can be estimated using an empirical expression reported in the literature:
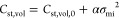
5where *C*_st,vol,0_ is the capacitance in the zero-charge limit and α is an empirical coefficient for the charge dependence of Stern capacitance.

Assuming that the electrode is saturated at the end of the charge half-cycle, there is no transport of ions across the spacer channel and the electrode. At equilibrium, the sum of Δϕ_D_ and Δϕ_s_ equals half of the potential across the cell for symmetric cells:
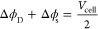
6

Combining eq 1 with eq 6, we can obtain the equilibrium charge density and salt concentration in the macropore under a given applied voltage. ES can be operated using many different schemes. For simplicity, we choose to analyze an ES process where charging is performed using a constant voltage until saturation and discharging is performed via short circuiting (i.e., zero voltage discharge).

### Simplified 1-D Dynamic ES Transport Model

To obtain the concentration profile in the diluate channel as a function of time, we employ a simplified 1-D dynamic ES transport model.^2,30^ The ion flux, *J*_i_, through the macropores is given by the Nernst–Planck equation:
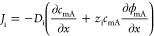
7where *D*_i_ is the diffusion coefficient, ϕ_mA_ is the macropore potential, and *x* is the coordinate starting from the spacer-electrode boundary to the electrode-current collector boundary (coordinate shown in Figure S1). Across the electrode, the mass balance of ion “i” can be written as
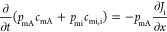
8where *p*_mA_ and *p*_mi_ are the macropore and micropore porosities and *c*_mi,i_ is the concentration of species i in micropores. The electrode potential is a constant, yielding:
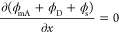
9

As ion transport leads to desalination in the spacer channel, the mass balance of salt can be written as
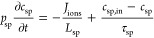
10where *p*_sp_ is the spacer channel porosity, *c*_sp_ is the spacer channel salt concentration, *L*_sp_ is the thickness of the spacer channel, τ_sp_ is the hydraulic retention time in the spacer channel, and *c*_sp,in_ is the spacer channel influent concentration. In the single-pass mode, *c*_sp,in_ is a constant and equals the feed concentration. In the semibatch mode with recirculation, *c*_sp,in_ is the concentration in the diluate or concentrate tank and can be expressed as
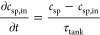
11where τ_tank_ is the hydraulic retention time in the tank. Lastly, we also consider ion flux and current continuities at the spacer–electrode interface:
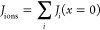
12
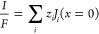
13

Solving eqs 1–13 yields the feed tank concentration, micropore charge density, and macropore salt concentration as a function of time. The specific energy consumption (SEC) of an ES process can be calculated using the following equation for short-circuit discharge where no energy is recovered:
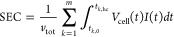
14where *v*_tot_ is the total volume of the dilute and concentrate water, *k* is the cycle number, *t*_*k*_,_0_ and *t*_*k*,hc_ are the initial and final time points of the *k*th charge half-cycle, *V*_cell_(*t*) and *I*(*t*) are the cell voltage and current at time *t*. Constant voltage in the charging stage is employed in this study. The final salt removal (SR) is obtained as
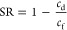
15where *c*_f_ and *c*_d_ are the concentrations of the feed and diluted stream, respectively. SR is equivalent to salt rejection in reverse osmosis or nanofiltration. The parameters utilized in the calculation are selected from ranges commonly reported or simulated in the literature, as listed in Table 1. These parameters in Table 1 are used throughout all simulations, except when the parameters are the subject of investigation.

**Table 1:** Parameters for Simulation

symbols	description	value	dimension	reference
*C*_st,vol,0_	zero-charge capacitance	200	F g^–1^	(31)
α	charge dependence of stern capacitance	20		(30)
*V*_cell_	cell voltage	1.2	V	(1)
ρ_elec_	electrode mass density	0.75	g mL^–1^	(32)
*p*_sp_	spacer porosity	0.5		(20)
*p*_mi_	microporosity	0.25		(33)
*p*_sp_	macroporosity	0.5		(34)
*L*_elec_	electrode thickness	250	μm	(35)
*L*_sp_	spacer thickness	250	μm	(36)
*T*_hc_	half-cycle time	30	min	(37)

### Mass Balance in ES with or without Mixing

In the dynamic ES transport model, the effect of mixing is studied by setting the initial boundary conditions of the macropore concentration for the charge and discharge half-cycles. Without mixing, the concentration in the macropores at the beginning of charge or discharge half-cycle equals the concentration of the diluate or the concentrate stream. With mixing, the concentration in the macropores at the beginning of the charge half-cycle equals the concentration of the brine, as the pores preserve the brine from the previous discharge half-cycle. Similarly, the concentration in the macropores at the beginning of the discharge half-cycle equals the concentration of the diluate.

In addition, analytical equations can be derived to relate the salt concentration in diluate, brine, the macropores, and micropores at the beginning of charge or discharge half-cycle and these concentrations at equilibrium with mass-balance equations. These analytical equations (eqs 16–19) are used to investigate the influence of mixing under different operational conditions and electrode properties on salt removal in a single-cycle batch mode due to their simplicity. Without mixing, the concentration in the macropores at the beginning of charge or discharge half-cycle equals the concentration of the feed. The mass balance holds for both charge half-cycle (eq 16) and discharge half-cycle (eq 17):
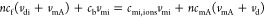
16
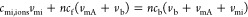
17where *n* is the van’t Hoff factor (e.g., *n* equals 2 for NaCl), *c*_f_ is the feed concentration, *v*_di_ is the volume of the diluate (in the flow channel, tubing, and the tank), and *v*_mA_ and *v*_mi_ are the volumes of electrode macropores and micropores, respectively. The product of concentration and volume at the beginning of the charge (or discharge) half-cycle (the left-hand side of eqs 16–19) equals the product of concentration and volume at the end of the charge (or discharge) half-cycle (the right-hand side of eqs 16–19). With mixing, we apply mass balance for the charge (eq 18) and discharge (eq 19) half-cycle:
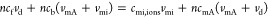
18
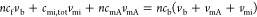
19where *c*_b_ is the brine concentration and *v*_b_ is the volume of the brine tank. The volumes of the macropores (*v*_ma_) and micropores (*v*_mi_) are given by
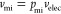
20
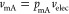
21
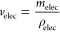
22where *p*_mi_ and *p*_mA_ are the microporosity and macroporosity and *v*_elec_, *m*_elec_, and ρ_elec_ are the electrode volume, mass, and density. Combining eqs 1–6 and 15–22, we can obtain the salt removal with or without mixing at a given operational conditions without solving the temporal and spatial evolution of concentration in the charge and discharge half-cycle in single-cycle semibatch mode.

### Single-Pass vs Semibatch Operation Modes in ES

**Figure 2:**
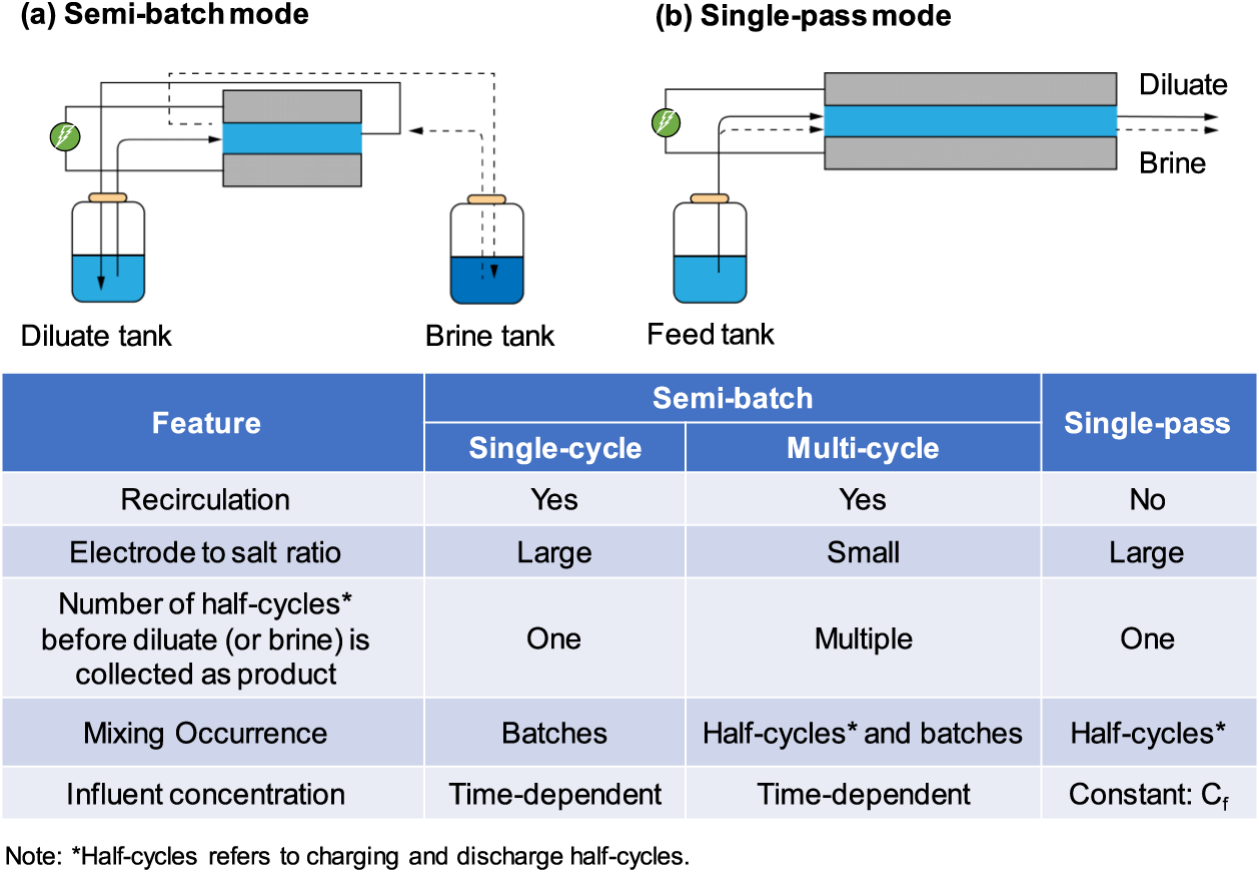
Schematics and feature comparison of (a) semibatch mode and (b) single-pass mode in ES. The semibatch mode can be further divided into a single-cycle semibatch mode and a multicycle semibatch mode, depending on the number of charge/discharge cycles a batch of solution is treated for before it is collected as the product freshwater.

Based on whether the solutions are recirculated between their respective tanks and the ES cell, we can categorize film electrode-based ES into two modes: semibatch and single-pass modes (Figure 2). In the semibatch mode, the diluate stream is recirculated between the ES cell and the diluate tank, whereas the brine stream is recirculated between the ES cell and the brine tank. Semibatch ES can have one or multiple charge–discharge cycles depending on the desalination goal and the operational parameters. Semibatch ES with a single flow cycle represents a process where the diluate stream is recirculated until the end of a charge cycle and then collected as the product water (Figure 2a). This is practical only if a small salinity reduction needs to be achieved or if a large electrode mass with high salt adsorption capacity is used to treat a small volume of feed solution. Alternatively, a semibatch ES can also be operated using multiple charge–discharge cycles where the diluate desalinated in previous charge cycles continues to be desalinated in subsequent charge cycles after the electrodes are regenerated in the discharge cycles. Only when the salinity of the diluate reaches a sufficiently low target level will the diluate be collected as the product water (Figure 2a).

In the single-pass mode, the feed solution flows through the ES cell once and flows out as the diluate, as the ions are removed from the solution and stored in the electrode temporarily. When the electrode is charged to the desired level (e.g., saturated at an applied voltage, or voltage becoming too high at an applied current), the cell is either short-circuited or a reverse voltage is applied to release the stored ions to the feed solution, resulting in a brine stream (Figure 2b). The key feature of the single-pass mode is that the effluents in charge or discharge half-cycles are not sent back to the tanks from which their respective influents are drawn from. For an ES process with a single-pass mode to generate a significant salinity reduction (from the feed solution to diluate), it requires a long hydraulic residence time and a relatively large electrode mass with a high salt adsorption capacity for a given volume of solution desalinated.

## Results and Discussion

### Impact of Mixing on Desalination Performance in Single-Cycle Semibatch Mode

**Figure 3:**
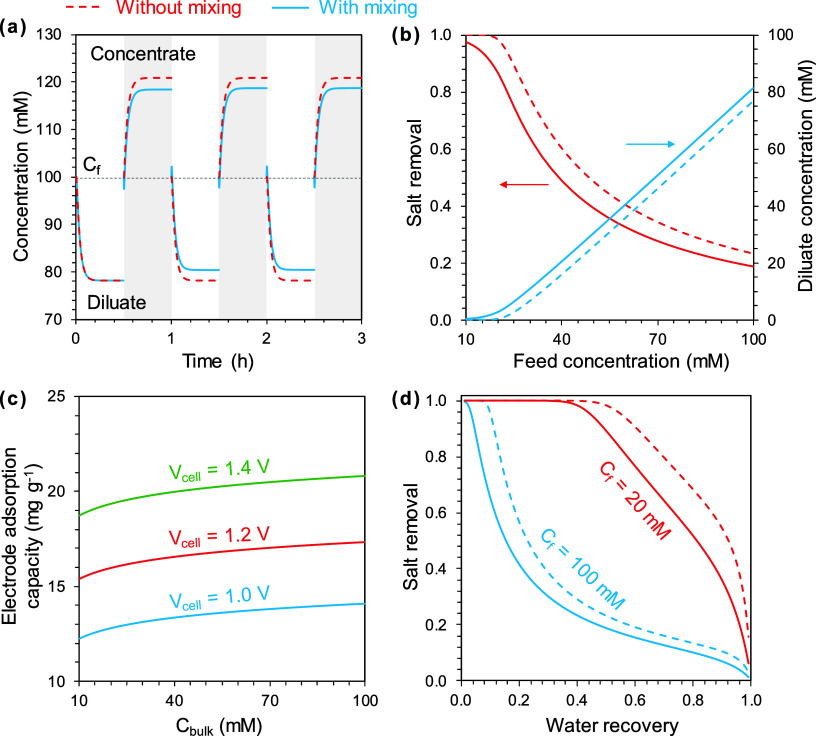
(a) Concentration of diluate and brine as a function of time in an ES process with single-cycle semibatch mode without mixing (red dashed curve) or with mixing (blue solid curve). (b) Influence of mixing on ES performance with varying feed concentration in single-cycle semibatch mode. (c) Dependence of electrode adsorption capacity on cell voltage and bulk salinity. (d) Influence of mixing on ES performance with varying water recovery in single-cycle semibatch mode. The results are simulated using the following parameters: the feed concentration is 100 mM (a) and the electrode loadings, defined as electrode mass per feed volume, are 100 g L^–1^ (a, c, d). Other parameter settings can be found in Table 1.

In the single-cycle semibatch mode, a large electrode mass (or area) is necessary to reach target salinity reduction, which in turn retains a large volume of solution in the macropores having the effluent salinity at the end of the preceding half-cycle. At the beginning of the charge half-cycle, the feed solution entering the flow channel experiences a concentration jump due to the mixing between the feed solution and the brine remaining in the macropores at the end of the discharge half-cycle. Similarly, at the beginning of the discharge half-cycle, mixing occurs between the influent with the feed salinity and the diluate remaining in the macropores at the end of the preceding charge half-cycle, which results in a sudden decrease in the concentration of the brine flow. These effects are evident when comparing the salinity of the solution in the channel at the beginning of the charge and discharge half-cycles to the influent salinity (Figure 3a). With the parameters used in this investigation, the presence of mixing reduces the salt removal from 40% to 32% for a 100 mM NaCl solution (typical brackish water salinity).

The impact of mixing on the ES performance varies with operational conditions and electrode properties. With other parameters kept constant, the salt removal decreases with an increasing feed concentration. An ES system that can achieve near complete desalination of a low-salinity feed solution (e.g., 10 mM) can only achieve a salt removal of ∼20% when the feed salinity increases to 100 mM (Figure 3b). Compared to the ideal scenario without mixing, salt removal is systematically undermined by mixing due to solution switch. The salt removal with mixing is around 80% of that without mixing with a feed concentration of 30–100 mM (Figure S2). At adsorption equilibrium, the electrode adsorption capacity increases with increasing bulk solution concentration and increasing the applied voltage also improves the electrode adsorption capacity (Figure 3c). However, the electrode capacity is only tens of milligrams of salt per gram of the AC electrode under commonly used operation parameters,^38^ which explains the relatively low salt removal for a feed even with a moderate salinity (e.g., 100 mM, as shown in Figure 3b).

The analysis shown in Figure 3b assumes a 50% water recovery (WR), defined as the ratio between the diluate volume and the total volume of the diluate and brine. With a low feed concentration (<20 mM), the salt removal first remains high (near 100%) with a WR below 40% as the electrodes have not reached saturation. Salt removal declines sharply when further increasing the WR due to electrode saturation. With a feed concentration of 100 mM, salt removal decreases with increasing WR (Figure 3d). With increasing WR, the volume of the diluate and thus the amount of salt to be removed increase, but the capacity of the electrode is fixed at a certain voltage. At the same time, these ions need to be concentrated in a smaller volume of brine, thus generating a more concentrated brine. This explains the trade-off between salt removal and WR. The presence of mixing further compromises salt removal by over 20% when WR exceeds 7% with a feed concentration of 100 mM (Figure S2).

**Figure 4:**
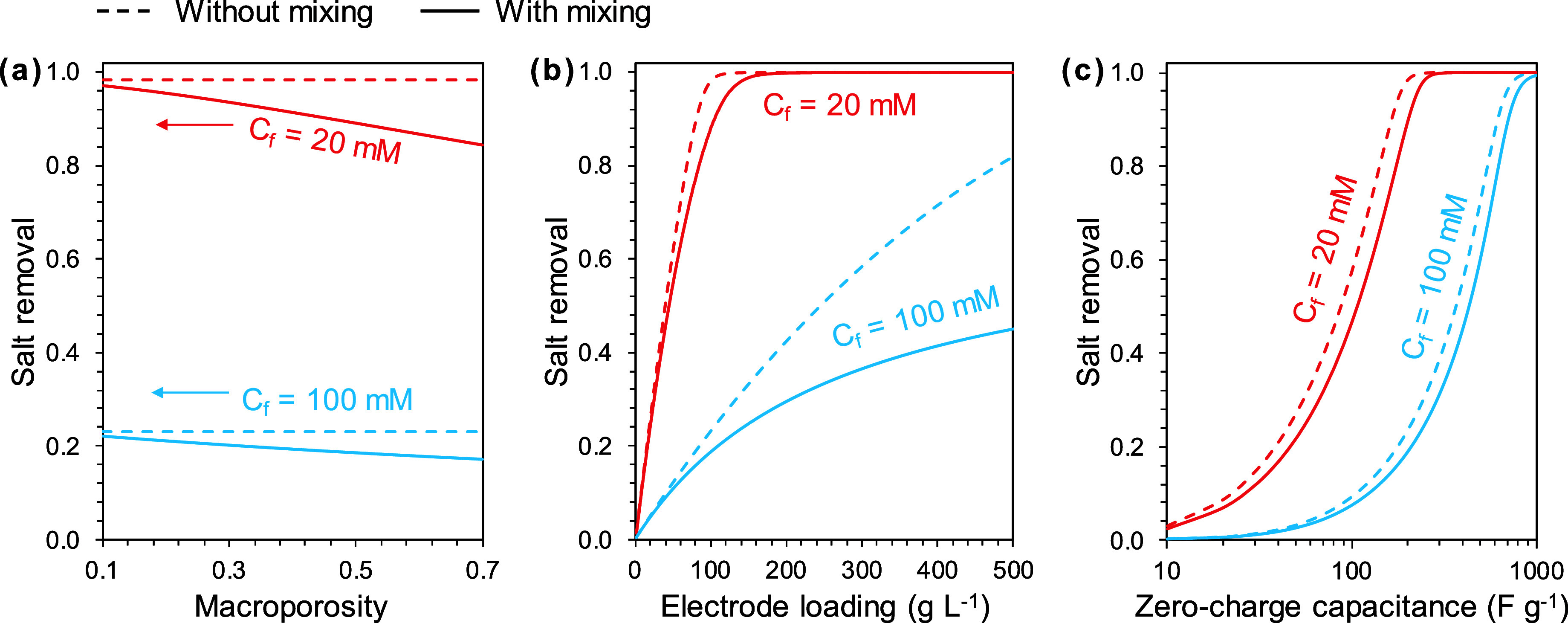
Influence of mixing on ES salt removal with varying macroporosity (a), electrode loading (b), and electrode Stern capacitance at zero-charge limit (c) in single-cycle semibatch mode. The impact was simulated under two feed concentrations: 20 and 100 mM. The electrode loading used in the calculation is 100 g L^–1^. Other parameter settings can be found in Table 1.

Without mixing, salt removal is unaffected by the macroporosity. With mixing, however, salt removal decreases with increasing macroporosity (Figure 4a), because a larger volume (relative to the spacer channel volume) of solution remaining in the macropores will mix with the influent solution. The percentage reduction of salt removal due to mixing also increases with an increasing microporosity (Figure S3). Despite this negative impact, the presence of macropores is necessary because they provide fast transport pathways for ions to access the micropores.^39^ Decreasing macroporosity will thus increase the ion transport resistance by reducing the “highways” for ions to transport from the spacer channel to the micropores.

To enhance salt removal, one effective strategy is to increase the electrode mass (or area) relative to the treated water volume. This strategy is generally feasible without considering the influence of mixing (Figure 4b). The salt removal of a 100 mM feed solution can be elevated to above 80%, though with an impractically large electrode to solution ratio (e.g., 500 g of electrode per liter of feed solution). However, in the presence of mixing during solution switch, even the benefit of using more electrodes is compromised due to the increased macropore volume. In other words, although a large electrode mass can store more salts per charge half-cycle, it also carries in the macropores a larger volume of solution that later becomes available for mixing during solution switching and thus cannot mitigate the negative impact of mixing. With 500 g of electrode for treating 1 L of feed solution (100 mM), the salt removal is only 45% when the effect of mixing is considered, vs 82% in the ideal scenario without mixing.

Increasing the electrode capacity effectively improves salt removal but cannot eliminate the influence of mixing (Figure 4c). Carbon electrodes reported in the literature have zero-charge capacitances ranging from 50 to 500 F g^–1^,^40^ which can only desalinate low-salinity brackish water (e.g., 20–65 mM) but fail to produce freshwater from brackish water with higher salinity. Although much effort has been devoted to developing high-capacity electrodes in the past decade, achieving a salt removal of over 90% from 1 L of a 100 mM solution (NaCl) using 100 g of electrodes requires the electrode capacity to exceed 700 F g^–1^. Using ES for seawater desalination is even more challenging and will require electrodes with capacity that is at least an order of magnitude higher than that of the state-of-the-art ones.

### Impact of Mixing on Desalination Performance in Multicycle Semibatch Mode

As shown in the previous section, a unit mass of electrode can store only a small number of ions during a charge half-cycle, which means an extremely large mass of electrode (per volume of solution) is needed to reach the target salt removal to desalinate brackish water even with a medium concentration. To address this challenge, multicycle semibatch mode has been employed in ES where the same feed solution (or brine) flows through the ES cell for multiple charge (or discharge) half-cycles, with salt removed (or released) incrementally each cycle to achieve a significant cumulative salt removal.^23,41^

**Figure 5:**
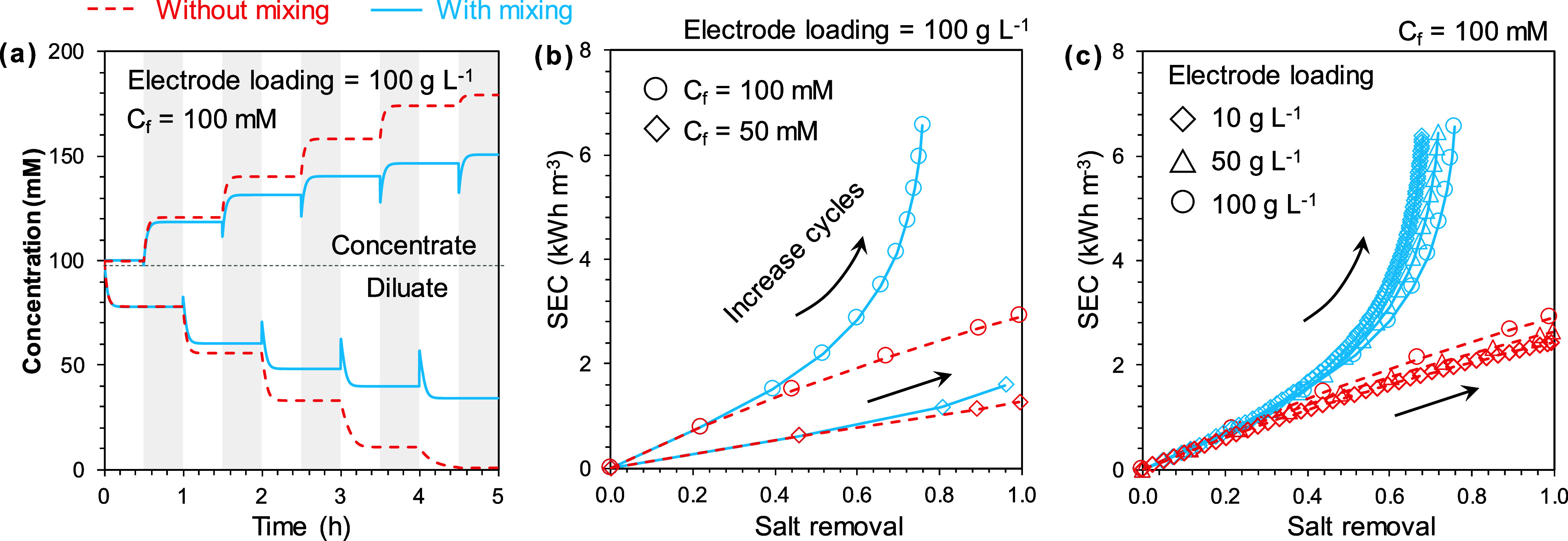
(a) Concentration of diluate and brine as a function of time in multicycle semibatch mode without mixing (red dashed curve) or with mixing (blue solid curve). The spikes in the blue curves represent the degree of mixing due to solution switch. (b, c) The relationship of specific energy consumption (SEC) and salt removal in multicycle semibatch mode without mixing (red dashed curve) and with mixing (blue solid curve) for (b) different feed concentrations (50 and 100 mM) and (c) different electrode loading (10, 50, and 100 g L^–1^). In both Figure 5b and Figure 5c, the increase in salt removal is achieved by increasing the charge/discharge cycles. The key parameters for simulation are shown in the figures, whereas the remaining parameters use values presented in Table 1.

In the multicycle semibatch mode, a small electrode mass can be used, which results in a small macropore volume as compared to the solution volume. However, the smaller relative macropore volume does not alleviate the detrimental impact of the mixing due to solution switch because it takes more cycles to achieve the target desalination, and the effect of mixing will build up over multiple cycles (Figure 5a). Without mixing, we can increase the number of cycles to achieve the desired level of salinity reduction. With mixing, however, the salt removal in each cycle decreases due to the increasingly severe mixing as the concentration difference between the brine and diluate grows. For example, the salt removal of a 100 mM NaCl solution after 5 cycles reaches 99% without mixing, whereas only 65% of salt is removed with mixing (Figure 5a). Mixing becomes increasingly severe as we achieve a more complete desalination (i.e., a larger difference between the diluate and brine concentrations), as evidenced by the growingly larger “spikes” observed at the solution-switch steps.

The influence of mixing on the salt removal and specific energy consumption (SEC) is further evaluated in multicycle semibatch mode. The salt can be fully removed from feed in the absence of mixing by increasing the number of charge–discharge cycles (red curves in Figure 5b,c). For example, with a constant electrode loading of 100 g L^–1^, the removal for a feed solution of 50 mM and 100 mM NaCl increases from 46% and 22% with a single cycle to over 99% after 3 cycles and 5 cycles without mixing (Figures 5b and S4). However, with mixing, the removal is only 96% and 65% after 3 cycles and 5 cycles for the same feed solutions (Figures 5b, S4).

The presence of mixing not only slows down the desalination rate but also limits the salt removal and increases SEC. Further increasing the cycle number from 5 to 10 only marginally increased the removal for a 100 mM NaCl solution from 65% to 75% though the SEC almost doubles (Figures 5b and S4). SEC without mixing is roughly linear to salt removal (Figure 5b,c), but SEC with mixing increases much more rapidly as salt removal increases, especially with a higher feed concentration. Though close to SEC and salt removal without mixing in the initial cycles, SEC and salt removal with mixing increasingly deviate from the ideal case as the cycle number increases. The presence of mixing during solution switch compromises the benefit of increasing cycle number in multicycle semibatch mode, which is anaglous to the reduced benefit of increasing electrode loading in single-cycle semibatch mode.

The SEC is similar for different electrode loadings to achieve the same salt removal (Figures 5c), again corroborating our previous observation that increasing the electrode loading cannot effectively mitigate the detrimental impact of mixing. Like the single-cycle semibatch mode, the benefit of elevating the salt removal by using a higher electrode loading in multicycle semibatch mode is also compromised due to increased macropore volume available for mixing. In summary, even though multicycle semibatch mode can achieve the same target level of desalination with less electrode loading as compared to the single-cycle semibatch mode, the impacts of electrode and system properties on the ES desalination performance due to the mixing effect are similar in both modes.

### Impact of Mixing on Salt Removal in Single-Pass Mode

**Figure 6:**
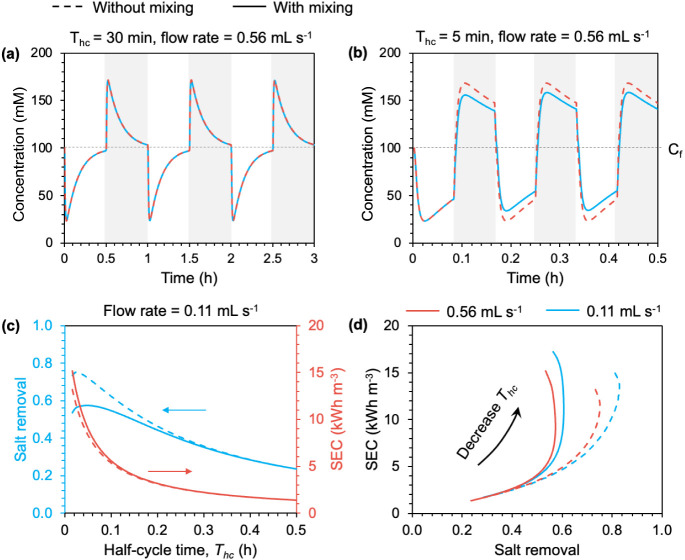
Concentrations of diluate and brine stream effluents as a function of time in single-pass mode without mixing (dashed curve) or with mixing (solid curve) with a half-cycle time (*T*_hc_) of (a) 30 and (b) 5 min. (c) Dependence of salt removal and SEC on half-cycle time without mixing (dashed curve) or with mixing (solid curve). (d) Relationship between SEC and salt removal as half-cycle time varies for two different flow rates, with and without mixing. The results are simulated all using 100 mM NaCl as the feed solution. Other parameter settings can be found in Table 1.

In most laboratory-scale single-pass experiments performed using constant voltage charge and short-circuit discharge, the detrimental impact of the solution switch can be rightfully ignored because there is minimum mixing with such an operation. Specifically, in most of such experiments, charging is performed until the electrodes are saturated (e.g., adsorption equilibrium is reached), and the flow channel is flushed with feed solution without any salt removed. Therefore, there is no salinity difference between the solutions flowing through the ES cell before and after solution switch. In other words, the flow channel is effectively flushed by the feed solution toward the end of both the charge and discharge half-cycles when neither charge nor discharge is actively performed (Figures 6a).

In such a context, solution switch does not induce mixing, yet flushing itself reduces the salt removal in the charge half-cycle and results in more wasted water in the discharge half-cycle. For example, the cumulative salt removal of a 30 min half-cycle time is only 24%; in comparison, the cumulative salt removal is 66% without mixing with a half-cycle time of 5 min in a single-pass mode. Without extensive flushing, the impact of mixing between the remaining solution in macropores and the influent feed solution cannot be ignored. For example, mixing reduces the maximum salt removal for a feed solution of 100 mM NaCl from 66% to 56% with a half-cycle time of 5 min (Figures 6b).

For practical applications of ES with the goal of producing fresh water, charging should be performed at an optimum half-cycle time to obtain high removal with low SEC. Both salt removal and SEC increase with a decreasing half-cycle time with a fixed electrode mass and flow rate (Figures 6c). The adverse effect of mixing becomes more severe with increasing salt removal as the half-cycle time decreases and a higher salt removal is achieved, but the impact of mixing on salt removal is much more significant than that on SEC. Increasing the flow rate with the same electrode mass does not mitigate the impact of mixing on salt removal when a relatively high salt removal is achieved with a very short half-cycle time (Figures 6d). In other words, in a practical single-pass ES system with a realistic electrode mass per volume of treated water, there is no viable means of achieving high salt removal if the feed salinity is moderately high. If we use a long half-cycle time, the effect of mixing is mitigated by extensive flushing, but the salt removal is low due to the long half-cycle; if we use a short half-cycle, which supposedly yields a high salt removal in the ideal scenario without mixing, mixing then plays an important role, which reduces the salt removal. We note that the ES with constant current operation will experience similar mixing due to solution switch as that situation described in Figure 6b and the short half-cycle time regime in Figure 6d.

### Why Is Mixing Typically Not a Concern in Conventional Fixed-Bed Adsorption

Depending on whether regeneration is performed, mixing may also be present in other fixed-bed adsorption processes based on granular AC, mineral adsorbents, and ion exchange resins. However, mixing is a unique challenge to ES because the equivalent bed-volume throughput of ES is orders of magnitude smaller than that of the typical fixed-bed adsorption processes. Bed-volume throughput is the number of bed volumes treated by an adsorption system before the adsorbents are saturated, or the ratio between throughput volume and bed volume. For instance, well-designed granular AC and ion exchange columns have a bed-volume throughput ranging from thousands to tens of thousands.^42−45^ In comparison, the equivalent bed-volume throughput for ES processes is mostly less than 10 and can fall below 1 when feed salinity is high (Section S1 and Figure S5). Therefore, the detrimental impacts of mixing on performance are significant in ES but not in conventional fixed-bed adsorption in which the volume of the affected solution is negligible compared to the treated volume.

The major difference in bed-volume throughput between fixed-bed adsorption processes and ES is mainly attributable to the difference in adsorbate concentration: in contaminant removal, the adsorbate concentration is relatively at the order of nano- to micro-molar,^42,46,47^ whereas in desalination, the concentration of adsorbate (i.e., salt) is at the order of tens to hundreds of millimolar. Due to their large bed-volume throughputs, many conventional fixed-bed adsorption processes do not even require adsorbent regeneration.^45,46^ Disposable adsorbents are practical in these fixed-bed adsorption systems due to the low cost of adsorbent per volume of treated water, which is certainly not the case for ES processes.

## Implications

Due to the limited adsorption capacity of film electrodes, ES systems require solution switch coupled with short-circuiting or voltage reversal to sustain desalination. The model-based analysis performed here reveals the following insights:

- Regardless of the single-cycle semibatch mode, multicycle semibatch mode, or single-pass mode (without extensive flushing), mixing between the influent solution and solution remaining in the electrode macropores leads to appreciable performance deterioration.
- The detrimental impacts on performance due to mixing are manifested by reduced salt removal and/or increased specific energy consumption. Such detrimental impacts are more severe with a larger difference between the diluate and brine concentrations, which occurs when the feed salinity is higher, the salinity reduction is more significant, and/or the water recovery is greater.
- Merely increasing the electrode loading per volume of solution treated is ineffective in mitigating the detrimental impacts of mixing. Increasing the specific adsorption capacity of electrodes helps alleviating the detrimental impacts of mixing. While research on ES has been extensively devoted to developing electrodes with higher capacity,^48,49^ the analysis presented in this work reveals that the true importance of a high specific adsorption capacity in desalination performance is mitigating the adverse impact of mixing due to solution switch.

In addition to desalination, research efforts have been growing to unlock new opportunities for ES in other applications, such as mineral extraction and resource recovery. For example, ES based on Li-intercalation electrode materials has been shown to be capable of highly selective separation with feed solutions of complex composition and high salinity, which makes ES a promising technological candidate for chemical-free direct lithium extraction.^14,15^ Film electrode-based ES for Li extraction also faces the challenge of mixing due to solution switch, which has additional detrimental impacts on selectivity because macropores store ions nonselectively and will release unwanted ions to the receiving solution.^25^

A common approach to minimize the adverse effects of mixing on selectivity is to add a rinsing step before a solution switch to remove solutions stored in the macropores. Such a rinsing step can improve the purity of lithium in the receiving solution but at the cost of more freshwater consumption. Addressing the negative impacts of solution switch in various applications of ES can benefit developing electrodes with higher capacity but only to a limited extent. ES systems employing flow electrodes are considered quasi-continuous. However, to obtain pure target substances in such systems, solid–liquid separation and rinsing are necessary to remove residual impurities from the flow electrode slurry. Future research should explore new configurations and operational modes to mitigate the detrimental impacts of mixing due to solution switch.

## Supporting Information Available

Schematic of the coordinate used in the 1-D dynamic CDI transport model (Figure S1); the ratio of salt removal with mixing and without mixing as a function of feed concentration and water recovery in one-cycle semibatch mode (Figure S2); the ratio of salt removal with mixing and without mixing as a function of macroporosity and electrode loading, and electrode capacity in one-cycle semibatch mode (Figure S3); the relationship of specific energy consumption (SEC) and salt removal as a function of cycle number in multicycle semibatch mode without and with mixing (Figure S4); estimation of bed-volume throughput for ES (Section S1 and Figure S5) (PDF)
