# Supplementary material for: Mixing due to Solution Switch Limits the Performance of Electrosorption for Desalination
Source: Environ Sci Technol. 2024 Jul 18;58(31):13995–4004. doi: 10.1021/acs.est.4c02681 (PMC11308525; doi:10.1021/acs.est.4c02681)
Supplement: Supplementary file 1 — es4c02681_si_001.pdf [file es4c02681_si_001.pdf]

Supporting information

Mixing due to Solution-switch Limits the Performance of Electro-sorption for Desalination

Environmental Science & Technology

Weifan Liu[a], Longqian Xu[a *], Zezhou Yang[b], Xudong Zhang[a], Shihong Lin[a, b *]

[a] Department of Civil and Environmental Engineering, Vanderbilt University, Nashville, Tennessee 37235-1831, USA

[b] Department of Chemical and Bimolecular Engineering, Vanderbilt University, Nashville, Tennessee 37235-1831, USA

corresponding author emails:

Longqian Xu: longqian.xu@vanderbilt.edu

Shihong Lin: shihong.lin@vanderbilt.edu

Contents:

5 Pages

1 supporting text, 5 Supporting Figures,

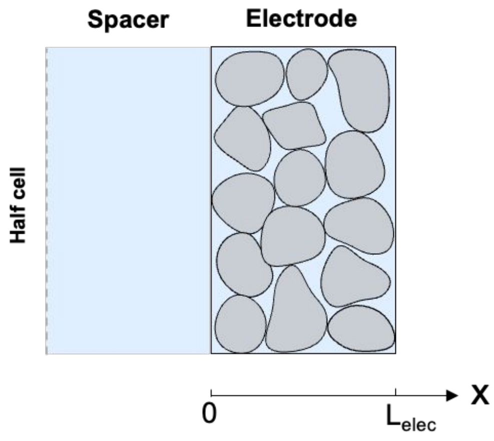

Figure S1. Schematic of the coordinate used in the 1-D dynamic CDI transport model. x = 0 at the spacer channel and electrode interface. x = L_{elec} at the electrode current collector boundary.

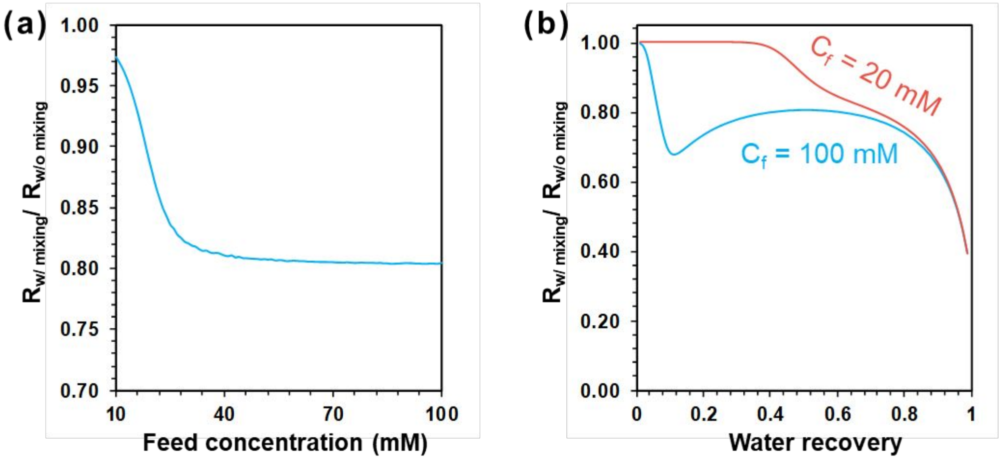

Figure S2. The ratio of salt removal with mixing and without mixing as a function of feed concentration (a) and water recovery (b) in one-cycle semi-batch mode. The electrode loadings used in the calculation is 100 g L^{-1}. Other parameter settings can be found in table 1.

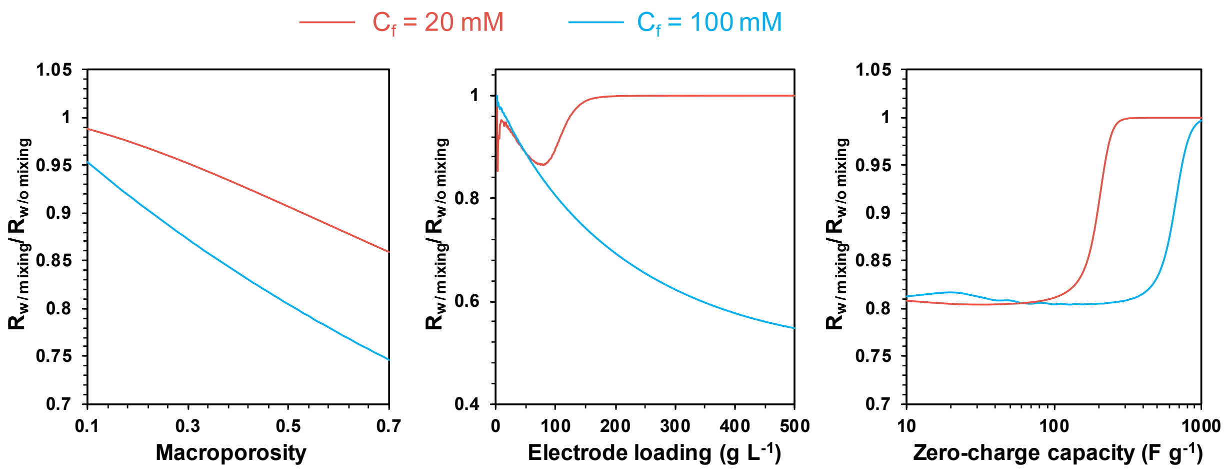

Figure S3. The ratio of salt removal with mixing and without mixing as a function of macroporosity (a) and electrode loading (b), and electrode capacity (c) in one-cycle semi-batch mode. The impact was simulated under two feed concentrations: 20 mM and 100 mM. The electrode loadings used in the calculation is 100 g L^{-1}. Other parameter settings can be found in table 1.

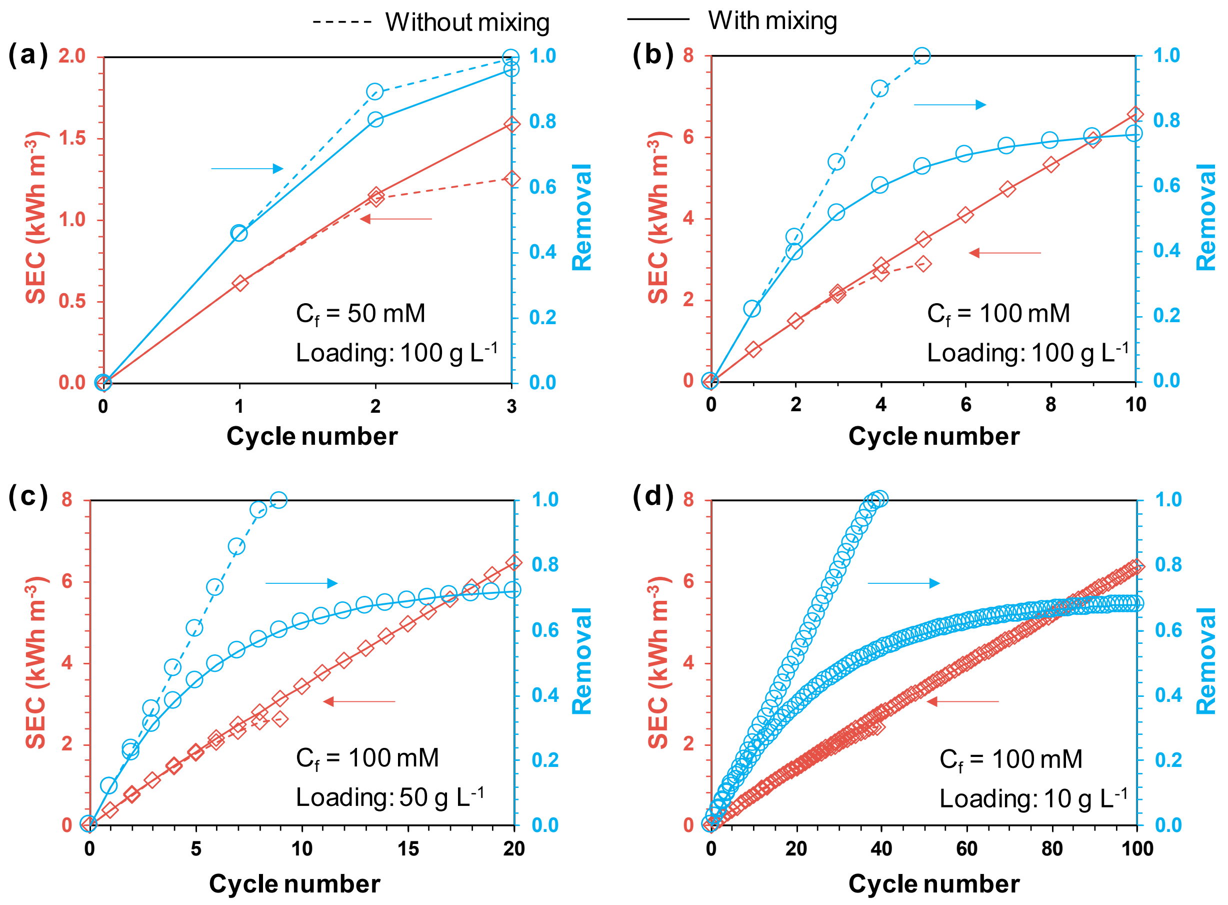

Figure S4. The relationship of specific energy consumption (SEC) and salt removal as a function of cycle number in multi-cycle semi-batch mode without (dashed curve) and with mixing (solid curve). Modeling parameters: (a) C_f= 50 mM, electrode loading = 100 g L^{-1}; (b) C_f= 100 mM, electrode loading = 100 g L^{-1}; (c) C_f= 100 mM, electrode loading = 50 g L^{-1}; (d) C_f= 100 mM, electrode loading = 10 g L^{-1}.

S1. The bed volume (BV) throughput of electro-sorption for desalination. The bed volume (BV) throughput defines the amount of water treatable before resin or absorbent regeneration is necessary. It inversely correlates with the level of mixing during solution switch. For electro-sorption desalination, the BV throughput is estimated using the equation:

$$\text{BV throughput} = \frac{\rho_{elec} C}{(c_f - c_d)}$$

where ρ_{elec} and C are the electrode density and capacity and are estimated to be 0.75 g ml^{-1} and 15 mg g^{-1} according to commonly reported values in literature. c_f and c_d are the feed concentration and diluate concentration. Setting the target diluate concentration as freshwater threshold of 500 mg L^{-1}, we can obtain the BV throughput under different feed concentrations (Fig. S5). Notably, the BV throughput for electro-sorption in desalination applications is generally under 10, and can be less than 1 for high-salinity feeds. In contrast, conventional fixed-bed adsorption methods typically achieve BV throughputs ranging from thousands to tens of thousands, which renders the detrimental impacts of mixing negligible.

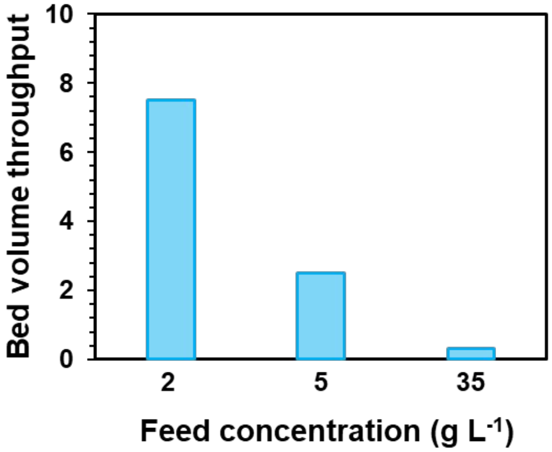

Figure S5. Bed volume throughput of electrosorption desalination with different feed concentrations. The electrode density and capacity 0.75 g ml^{-1} and 15 mg g^{-1}. The target diluate concentration is 500 mg L^{-1}.
